# A MYC-Driven Plasma Polyamine Signature for Early Detection of Ovarian Cancer

**DOI:** 10.3390/cancers13040913

**Published:** 2021-02-22

**Authors:** Johannes F. Fahrmann, Ehsan Irajizad, Makoto Kobayashi, Jody Vykoukal, Jennifer B. Dennison, Eunice Murage, Ranran Wu, James P. Long, Kim-Anh Do, Joseph Celestino, Karen H. Lu, Zhen Lu, Robert C. Bast, Samir Hanash

**Affiliations:** 1 Department of Clinical Cancer Prevention, The University of Texas M. D. Anderson Cancer Center, Houston, TX 77030, USA; jffahrmann@mdanderson.org (J.F.F.); dm11018s@st.kitasato-u.ac.jp (M.K.); jvykouka@mdanderson.org (J.V.); jbdennis@mdanderson.org (J.B.D.); ENMurage@mdanderson.org (E.M.); RWu2@mdanderson.org (R.W.); 2 Department of Biostatistics, The University of Texas M. D. Anderson Cancer Center, Houston, TX 77030, USA; EIrajizad@mdanderson.org (E.I.); JPLong@mdanderson.org (J.P.L.); kimdo@mdanderson.org (K.-A.D.); 3 Department of Gynecological Oncology and Reproductive Medicine, The University of Texas M. D. Anderson Cancer Center, Houston, TX 77030, USA; jcelesti@mdanderson.org (J.C.); khlu@mdanderson.org (K.H.L.); zlu@mdanderson.org (Z.L.); 4 Department of Experimental Therapeutics, The University of Texas M. D. Anderson Cancer Center, Houston, TX 77030, USA; rbast@mdanderson.org

**Keywords:** blood-based biomarkers, polyamines, ovarian cancer, early detection

## Abstract

**Simple Summary:**

There is a need for additional marker(s) to detect early-stage ovarian cancer that would augment the performance of CA125. Herein, we report a polyamine signature that is detected in the blood and that has value for detecting ovarian cancers at an early stage. The polyamine signature was able to complement CA125 in identifying more ovarian cancer cases that would have been missed by CA125 alone. Our validation of a polyamine signature provides compelling evidence for the value of blood polyamine metabolites as markers for ovarian cancer detection.

**Abstract:**

MYC is an oncogenic driver in the pathogenesis of ovarian cancer. We previously demonstrated that MYC regulates polyamine metabolism in triple-negative breast cancer (TNBC) and that a plasma polyamine signature is associated with TNBC development and progression. We hypothesized that a similar plasma polyamine signature may associate with ovarian cancer (OvCa) development. Using mass spectrometry, four polyamines were quantified in plasma from 116 OvCa cases and 143 controls (71 healthy controls + 72 subjects with benign pelvic masses) (Test Set). Findings were validated in an independent plasma set from 61 early-stage OvCa cases and 71 healthy controls (Validation Set). Complementarity of polyamines with CA125 was also evaluated. Receiver operating characteristic area under the curve (AUC) of individual polyamines for distinguishing cases from healthy controls ranged from 0.74–0.88. A polyamine signature consisting of diacetylspermine + N-(3-acetamidopropyl)pyrrolidin-2-one in combination with CA125 developed in the Test Set yielded improvement in sensitivity at >99% specificity relative to CA125 alone (73.7% vs 62.2%; McNemar exact test 2-sided P: 0.019) in the validation set and captured 30.4% of cases that were missed with CA125 alone. Our findings reveal a MYC-driven plasma polyamine signature associated with OvCa that complemented CA125 in detecting early-stage ovarian cancer.

## 1. Introduction

Currently, over 70% of patients with ovarian cancer present with advanced-stage (III-IV) disease, with dismal 5-year survival rates of less than 30%. Survival rates up to 70–90% can, however, be achieved with conventional surgery and chemotherapy, when disease is localized to the ovary (stage I) or pelvis (Stage II). [1,2] To-date, CA125 is the most investigated ovarian cancer early detection marker. [3] Neither CA125 nor transvaginal sonography (TVS) alone has adequate sensitivity or specificity for early detection. A two-stage strategy whereby rising CA125 prompts TVS in a limited fraction of women screened can achieve adequate specificity, but the sensitivity of CA125 is limited. [4] Moreover, only 80% of epithelial ovarian cancers express significant levels of CA125. [5] Thus, there is a need for an additional marker(s) to detect early-stage disease that would complement the performance of CA125.

Ovarian cancer and triple-negative breast cancer (TNBC) share common genomic features including MYC copy-number amplification. [6] MYC is an oncogenic driver in the pathogenesis of ovarian cancer. [7,8,9] We have previously demonstrated that MYC regulates the transcription of several polyamine metabolizing enzymes (PMEs) in triple negative breast cancer and that a plasma polyamine signature is associated with TNBC development and progression. [10] Herein, we tested whether a plasma polyamine signature would similarly be associated with ovarian cancer. We also evaluated whether polyamines, in combination with CA125, would improve classification performance compared to CA125 alone. 

## 2. Materials and Methods

### 2.1. Blood Samples

EDTA-plasma samples were obtained from the Normal Risk Ovarian Cancer Screening Study (NROSS) and stored at the MD Anderson Gynecologic Cancer Bank. All biospecimen were processed at a central site using a standardized protocol; EDTA-plasmas were stored in −80 °C until use. Ethical approval was obtained for these samples from the appropriate institutional review boards/ethic committees at MD Anderson and collaborating institutions under IRB protocol LAB04-0687. All participants had consent for the use of samples in ethically approved secondary studies.

Randomly selected healthy control plasmas were obtained from the NROSS cohort. Over the last two decades, the NROSS trial has involved 6379 postmenopausal women over the age of 50 at average risk for developing ovarian cancer and who have been followed with annual CA125 measurements and who have been referred for transvaginal ultrasound and gynecological evaluation if CA125 values increase from each individual’s baseline as judged by the Risk of Ovarian Cancer Algorithm (ROCA). Healthy controls were at least 12 months postmenopausal, between the ages of 50–75 and followed for a minimum of 7 years to ensure cancer-free status; cancer-free status was based on an annual self-reported questionnaire.

Plasma samples from treatment-naïve cases and control subjects presenting with benign pelvic masses were drawn from the MD Anderson Gynecologic Cancer Bank. Cases were randomly selected and not prioritized based on CA125 values.

Initial testing was performed using plasmas from 41 early stage (I-II) cases, 75 late-stage (III-IV) cases, 71 healthy controls and 72 patients with benign pelvic masses. The validation set consisted of an independent set of plasmas from 61 early-stage cases and 71 healthy controls. Subject characteristics are provided in Table 1. Detailed information on the benign pelvic masses as well as histological subtype of ovarian cancers amongst cases is provided in Table A1 in the Appendix A.

### 2.2. Metabolomics Analysis

Detailed information is provided in the Appendix A. Semi-quantitative measurement of plasma polyamines was conducted on a Waters Acquity™ UPLC system with 2D column regeneration (I-class and H-class) coupled to a Xevo G2-XS quadrupole time-of-flight (qTOF) mass spectrometer. Chromatographic separation was performed using HILIC (Acquity™ UPLC BEH amide, 100 Å, 1.7 µm 2.1 × 100 mm) and C18 (Acquity™ UPLC HSS T3, 100 Å, 1.8 µm, 2.1 × 100 mm) columns at 45 °C. Mass spectrometry data were acquired in sensitivity, positive electrospray ionization mode. Acquisition was carried out with instrument auto-gain control to optimize sensitivity during sample acquisition; data processing was performed as previously described [10,11].

Four polyamines (diacetylspermine (DAS), acetylspermidine (AcSpmd), diacetylspermidine (DiAcspmd), and N-(3-acetamidopropyl)pyrrolidin-2-one (N3AP)) were detected and quantified in plasmas of cases and controls (Table A2 in the Appendix A). Coefficient of variation (CV) values for the measured polyamines in quality controls are provided in Table A3 in the Appendix A. On average, CV values for measured polyamines in quality control samples were below 13%.

### 2.3. Measurement of CA125 Levels

Automated immunoassay kits for determining the concertation of CA125 antigen were purchased from Roche Diagnostics USA (Indianapolis, IN, USA).

### 2.4. Statistical Analyses

For two-class comparisons, statistical significance was determined using the Wilcoxon rank sum test. Statistical significance was determined at *p*-values <0.05. Receiver operating characteristic curves were generated using R (R version 3.6.0). The 95% confidence intervals presented for the individual performance of each biomarker were based on the bootstrap procedure in which we re-sampled with replacement separately for the controls and the diseased 1000 bootstrap samples. To identify the optimal combination of markers amongst CA125 and four other polyamines, we implemented Lasso regression. [12] Ten-fold cross-validation on the training dataset with AUC as the measurement of classification performance was incorporated to find the best tuning parameters (λ). This parameter yielded CA125, DAS, and N3AP as the best combination of biomarkers; a biomarker panel using CA125, DAS, and N3AP were subsequently derived using a logistic regression model. The estimated AUC of the proposed metabolite panel was derived by using the empirical ROC estimator of the linear combination corresponding to the aforementioned model. 

Confusion matrices were utilized to describe the classification model of the 3-marker panel or CA125 alone at a 99% specificity cutoff. Rows of the matrix display the predicted classes (case or control) whereas columns represent the actual classes (case or control). 

To test whether the 3-marker panel yielded statistically significant classifier improvements over CA125, the McNemar exact test was applied to compare two binomial proportions for patients with two different biomarker scores [13]. Herein, a 2 × 2 table was generated wherein the first cell represents the number of patients that both markers predict correctly (a), the second one represents the number of patients correctly identified by CA125 and misclassified by the 3-marker panel (b), the third one represents the number of patients misclassified by CA125 but correctly identified by the 3-marker panel (c) and the last cell represents the number of patients misclassified by both markers (d). Therefore, the null and alternative hypothesize are as follows:

H_0: P_b = P_c

H_a: P_b < P_c

Herein, P_i denotes the probability of occurrence in cell i. An exact binomial test was used to achieve *p*-value [14].

All statistical tests were two-sided unless specified otherwise.

## 3. Results

### 3.1. Polyamine Levels in Plasma of Ovarian Cancer Patients and Model Development

To determine whether a polyamine signature is associated with ovarian cancer, we screened polyamine levels in plasma from 116 OvCa patients (41 early stage + 75 late stage, 91 serous, 25 non-serous) and 71 healthy controls (Test Set) using ultrahigh performance liquid chromatography mass spectrometry (Table 1). We additionally evaluated the levels of polyamines in plasma from 72 patients presenting with diverse benign conditions to determine the specificity of polyamines for OvCa (Table 1; detailed information is provided Table A1).

**Figure 1:**
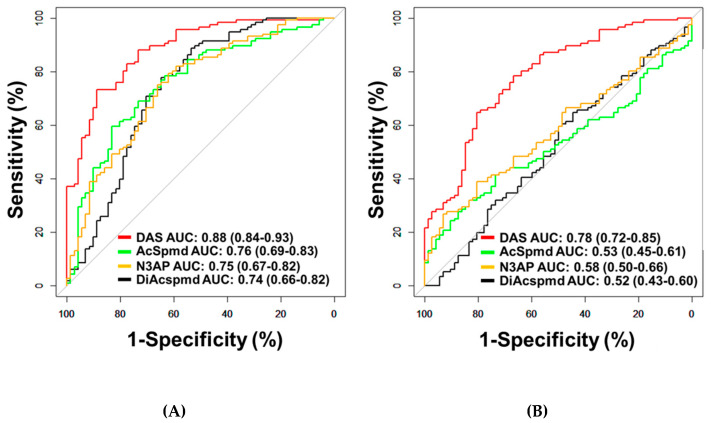
Classification performances of plasma polyamines in the Test Set. (**A,B**) Area under the curve (AUC) of DAS for delineating all cases (*n* = 116) from healthy controls (*n* = 71) (**A**) or patients with benign pelvic masses (*n* = 72) (**B**).

**Figure A1:**
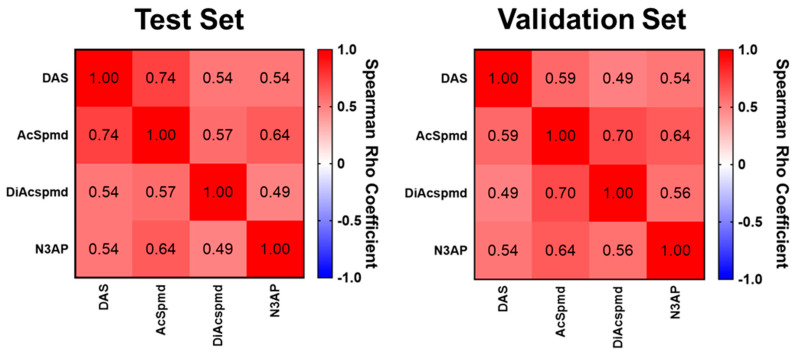
Spearman correlation matrix for polyamines. Heatmaps depicting spearman correlation coefficients amongst the 4 measured polyamines in the Test Set (left panel) and Validation Set (right panel). Embedded values represent Spearman correlation coefficients. All cases and controls corresponding to the respective test set were included in the analyses. Abbrev. DAS- diacetylspermine; AcSpmd- acetylspermidine; DiAcspmd- diacetylspermidine; N3AP- N-(3-acetamidopropyl)pyrrolidin-2-one.

**Figure A2:**
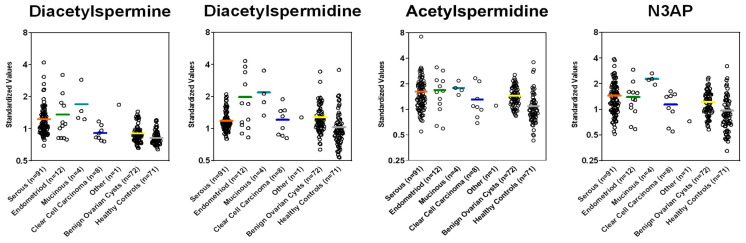
Plasma levels of polyamines in cases stratified by histology and controls in the Test Set.

A total of four polyamines (DAS, AcSpmd, DiAcspmd, and N3AP) were detected and quantified in plasmas of cases and controls (Table A2 in the Appendix A). AUCs of individual polyamines for distinguishing OvCa from healthy controls ranged from 0.74–0.88 (Figure 1A; Table A4 in the Appendix A). Measured polyamines were generally positively correlated to each other (Figure A1 in the Appendix A). Of the measured polyamines, DAS exhibited the highest classification performance for distinguishing ovarian cancer cases from healthy controls (AUC: 0.88 (95% C.I., 0.84–0.93)) or patients with benign pelvic masses (AUC: 0.78 (95% C.I., 0.72–0.85)) (Figure 1A,B; Table A4 in the Appendix A). AUCs of DAS for distinguishing all serous cases from healthy controls or subjects with benign pelvic masses were 0.90 and 0.80, respectively (Table A5 in the Appendix A). When considering only early-stage serous cases, DAS yielded AUCs of 0.81 and 0.70 in comparison to healthy controls or subjects with benign pelvic masses, respectively (Table A5 in the Appendix A). AUCs of DAS for distinguishing non-serous OvCa cases from healthy controls or subjects with benign pelvic masses or healthy controls were 0.83 and 0.71, respectively (Table A5 and Figure A2 in the Appendix A).

**Figure 2:**
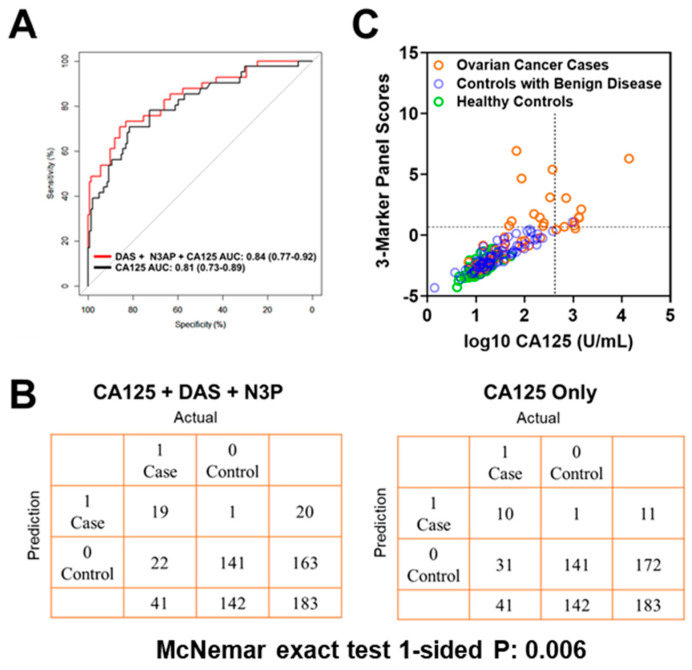
Classification performance of the 3-marker panel and CA125 in the Test Set. (**A**) Area under the curve for the 3-marker panel consisting of DAS + N3AP + CA125, and CA125 only. (**B**) Confusion matrix describing the performance of the classification model corresponding to the 3-marker panel and CA125 alone at 99% specificity. Statistical significance was determined by 1-sided McNemar exact test. (**C**) Scatter plot illustrating the distribution of the 3-marker panel scores (Y-axis) and log10 CA125 values (X-axis). Broken lines represent 99% specificity cutoffs.

Using logistic regression models, we determined whether the combination of polyamines plus CA125 would yield improved classification performance in differentiating early-stage OvCa cases from controls (healthy subjects + subjects with benign pelvic masses) when compared to CA125 alone. The combination of plasma DAS + N3AP + CA125 was identified as the best panel with an AUC point estimate of 0.84 (95% C.I., 0.77–0.92) (Figure 2A). Given the low prevalence of ovarian cancer (11.4 in every 100,000 people), a screening test must achieve high sensitivity at high specificity to avoid unacceptable levels of false-positive results. Therefore, we evaluated the sensitivity of the 3-marker panel (DAS + N3AP + CA125) in comparison to CA125 alone at corresponding specificities of 99%, 98.5% and 97% (Table A6 in the Appendix A). Compared to CA125 alone, the 3-marker panel yielded statistically significantly improved sensitivity at 99% and 98.5% specificity (1-sided McNemar Exact test P: 0.006 and 0.04, respectively) (Table A6 in the Appendix A). A confusion matrix describing the performance of the classification model corresponding to the 3-marker panel and CA125 alone at 99% specificity illustrates that the 3-marker panel correctly identified 19 out of 44 early-stage OvCa cases (46.3% sensitivity) whereas CA125 alone correctly identified 10 out of early-stage 41 OvCa cases (24.3% sensitivity) (1-sided McNemar exact test P: 0.006) (Figure 2B,C). 

In our study, correlation analyses between DAS, N3AP, and CA125 levels (which comprise the 3-marker panel) with ages amongst healthy controls were non-statistically significant (data not shown). Age-adjusted ROC analyses revealed that the 3-marker panel of DAS + N3AP + CA125 (age-adjusted AUC: 0.80 (95% CI: 0.71–0.88)) yielded improved classifier performance as compared to CA125 alone (age-adjusted AUC: 0.70 (95% CI: 0.58–0.82), Delong’s comparison of AUCs 1-sided P: 0.002). These findings imply that age is not a confounder in the context of the current study.

### 3.2. Model Validation in an Independent Cohort of Early Stage Ovarian Cancer Patients

**Figure 3:**
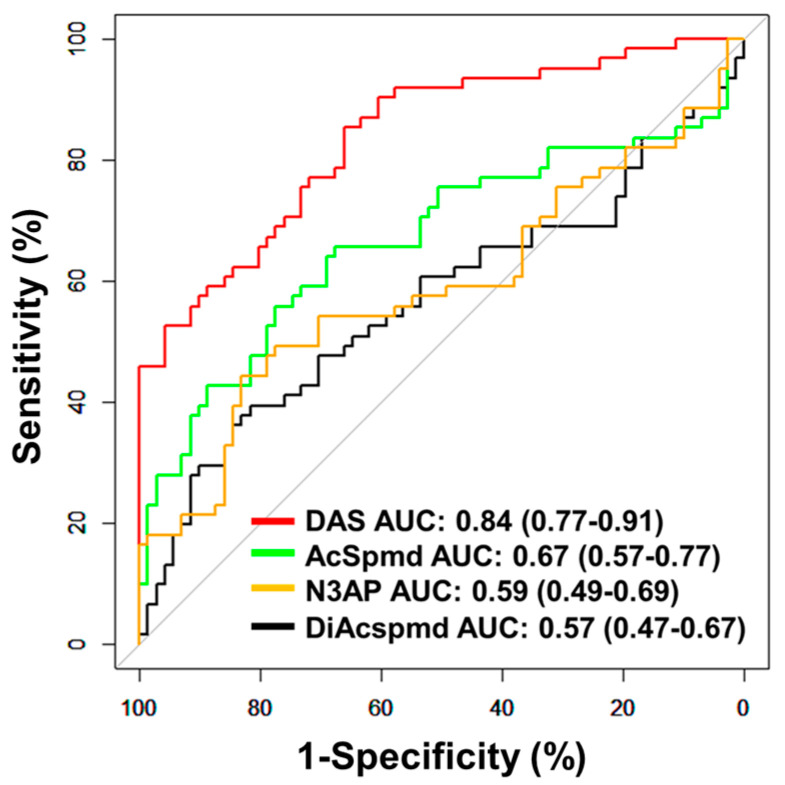
Classification performances of plasma polyamines in the validation set. The area under the curve (AUC) of individual polyamines for delineating all cases (*n* = 61) from healthy controls (*n* = 71).

**Figure A3:**
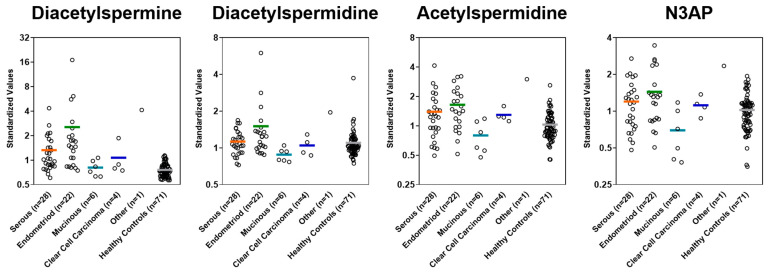
Plasma levels of polyamines in cases stratified by histology and controls in the Validation Set.

Further validation of the polyamine metabolites individually and the fixed 3-marker panel consisting of DAS + N3AP + CA125 was performed in an independent set of plasma samples consisting of 61 early-stage OvCa cases (serous = 28, non-serous = 33) and 71 healthy controls (Validation Set; Table 1). AUCs for individual polyamine metabolites ranged from 0.57–0.84 (Figure 3). Plasma DAS exhibited the highest AUC point estimate for delineating OvCa cases from controls (AUC: 0.84 (95% C.I., 0.77–0.91)) (Figure 3). Classification performance of plasma DAS in distinguishing serous (*n* = 28) and non-serous cases (*n* = 33) from healthy controls was 0.84 (95% C.I., 0.75–0.93) and 0.84 (95% C.I., 0.75–0.92), respectively (Table A7 and Figure A3 in the Appendix A).

**Figure 4:**
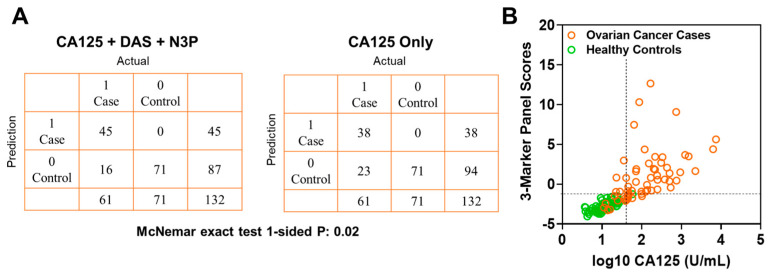
Classification performance of the 3-marker panel and CA125 in the validation set. (**A**) Confusion matrix describing the performance of the classification model corresponding to the 3-marker panel and CA125 alone at 99% specificity. Statistical significance was determined by 1-sided McNemar exact test. (**B**) Scatter plot illustrating the distribution of the 3-marker panel scores (Y-axis) and log10 CA125 values (X-axis). Broken lines represent >99% specificity cutoffs.

In the validation set, the fixed 3-marker panel yielded an AUC of 0.95 (95% C.I., 0.92–0.99) whereas CA125 yielded an AUC of 0.96 (95% C.I., 0.94–0.99). Sensitivity of the 3-marker panel using fixed beta-coefficients derived from the test set at corresponding specificities of >99%, 98.5%, and 97% for the validation set were 73.7, 78.6, and 83.6, respectively (Table A6 in the Appendix A). Compared to CA125 alone, the 3-marker panel yielded statistically significantly improved sensitivity at >99% specificity (3-marker panel sensitivity: 73.7%, CA125 sensitivity: 62.2%, 1-sided McNemar exact test P: 0.02) (Table A6 in the Appendix A). A confusion matrix describing the performance of the classification model corresponding to the 3-marker panel and CA125 alone at >99% specificity shows that the 3-marker panel correctly identified 45 out of 61 early-stage OvCa cases in the validation set whereas CA125 alone correctly identified 38 out of 61 early-stage OvCa cases, which corresponds to capturing 30.4% of cases missed by CA125 alone (Figure 4A,B). 

**Figure A4:**
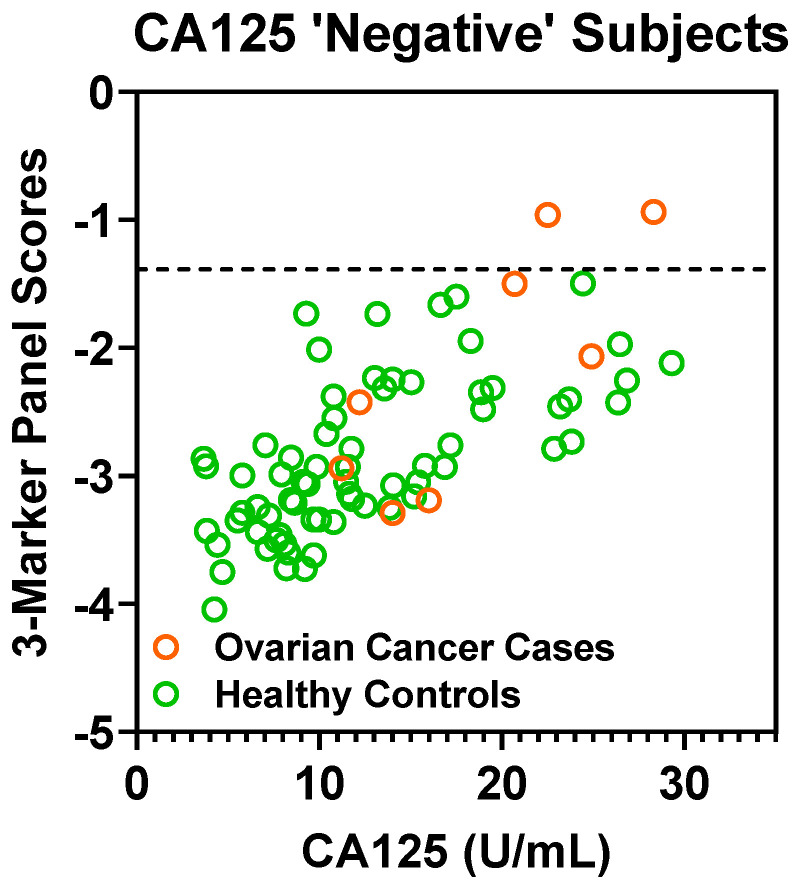
Scatter plot of depicting 3-marker panel scores (Y-axis) and CA125 values (X-axis) in CA125 ‘negative’ (defined as ≤35 U/mL) subjects in the Validation Set. Dashed lines represent 100% specificity cutoff.

A CA125 cutoff value of 35 U/mL is considered the upper limit of “normal” [15]. In the validation set, a cutoff value of 35 U/mL captured 53 out of 61 cases (86.9% sensitivity) with 2 false-positives (97.2% specificity). If considering cases “negative” for CA125 (defined as ≤ 35 U/mL), the fixed 3-marker panel identifies an additional 2 of the 8 early-stage cases (25.0% sensitivity) without any additional false-positives (Figure A4 in the Appendix A). Notably, these two additional cases identified by the 3-marker panel were stage I high-grade serous carcinomas.

## 4. Discussion

We demonstrated and validated that polyamines are statistically significantly elevated in plasmas of ovarian cancer cases in comparison to controls using two independent case-control cohorts. Of the measured polyamines, DAS exhibited the highest AUC for distinguishing cases from healthy controls or subjects presenting with benign pelvic masses. Importantly, we demonstrated that a 3-marker panel consisting of DAS + N3AP + CA125 yielded improved sensitivity at >99% specificity in comparison to CA125 only, resulting in capturing early-stage cases that were missed by CA125 alone in the validation set. Classifier performance of polyamines was higher in late-stage compared to early-stage cases, implicating that the polyamine levels reflect disease burden. Further, polyamines were elevated in both serous and non-serous cases. Recent reports indicated marked heterogeneity in clinical outcomes of ovarian cancer histological subtypes with non-serous subtypes exhibiting survival rates similar to or worse than that of high-grade serous carcinomas [16]. A blood test that can broadly identify ovarian cancers is, therefore, desirable.

There are potential limitations inherent to the available cohorts used in the study. Healthy controls tended to be older than cases. However, the 3-marker panel retained improved classifier performance compared to CA125 alone when adjusting AUCs for age, indicating that age is not a confounder variable in our analyses. Our study has a moderate sample size of early-stage OvCa cases. We emphasize that polyamines, DAS, in particular, yielded a good classifier performance for distinguishing early-stage OvCa cases from healthy controls in both the test and validation set. The performance of polyamine markers was reduced when considering subjects with benign pelvic masses suggestive that the presence of benign conditions may result in potential false-positives. Nevertheless, our findings demonstrated that polyamines, particularly DAS, were capable of distinguishing early-stage OvCa from subjects presenting with benign pelvic masses. 

Integrated genome analysis has reported that the *MYC* gene is amplified in 30–40% of human ovarian tumors [8,17,18,19]. In addition to *MYC*, copy-number amplification and/or overexpression of *MYCL1* and *MYCN* have also been reported in ovarian tumors [20,21,22]. We and others have demonstrated that the polyamine metabolizing enzymes ODC1, SRM and SMS are transcriptionally regulated by MYC [10,23,24,25] thereby providing a link between our polyamine signature and regulation via oncogenic MYC. In the context of our study, information regarding MYC status in tumors of subjects for which plasma polyamine levels were analyzed was not available thus precluding correlative analyses.

Prior studies have examined polyamine levels in urine and in one study, urine polyamines were found to be elevated among subjects with ovarian cancer. Similar to our findings, DAS exhibited the best classification performance for delineating ovarian cancer cases from individuals with benign disease and was found to be associated with disease progression [26].

In conclusion, we have identified an MYC-driven polyamine signature that reflects the early pathogenesis of ovarian cancer. Given the substantial interest in developing strategies for cancer detection and given the limited performance of CA125, our validation of a polyamine signature for early-stage ovarian cancer that complements CA125 provides supportive evidence for the utility of aberrant polyamine metabolism for cancer detection. Our findings provide a basis for the inclusion of the 3-marker panel on validation studies that encompass other biomarker candidates [26,27,28].

## Figures and Tables

**Table 1:** Patient characteristics for the Test Set and Validation Set.

Patient Characteristics for Specimen Sets	Test Set			Validation Set	
	Cases	Controls #1 †	Controls #2 ‡	Cases	Controls #1 †
Number of Subjects	116	71	72	61	71
Age (mean +/− stdev)	58 +/− 13	69 +/− 7	56 +/− 13	57 +/− 15	65 +/− 9
CA125 (u/mL), median (25th/75th percentile)	224 (87/561)	12 (9/16)	23 (13/59)	102 (45/321)	11 (8/17)
Serous					
Stage I, N (%)	11 (9.5)	-	-	13 (21.3)	-
Stage II, N (%)	5 (4.3)	-	-	15 (24.6)	-
Stage III, N (%)	64 (55.2)	-	-	-	-
Stage IV, N (%)	11 (9.5)	-	-	-	-
Non-Serous					
Endometrioid					
Stage I, N (%)	10 (8.6)	-	-	16 (26.2)	-
Stage II, N (%)	2 (1.7)	-	-	6 (9.8)	-
Mucinous					
Stage I, N (%)	3 (2.6)	-	-	6 (9.8)	-
Stage II, N (%)	1 (0.9)	-	-		
Clear Cell Carcinoma					
Stage I, N (%)	6 (5.2)	-	-	3 (4.9)	-
Stage II, N (%)	2 (1.7)	-	-	1 (1.6)	-
Other					
Transitional cell carcinoma (Stage I)	1 (0.9)	-	-	1 (1.6)	-

† Controls #1- Healthy Controls. ‡ Controls #2- Subjects with benign pelvic masses.

## Data Availability

Relevant data supporting the findings of this study are available within the Article and Appendix A or are available from the authors upon reasonable request.

## Appendix A

### Appendix A.1. Metabolomics Analysis

Plasma metabolites were extracted from pre-aliquoted EDTA plasma (10 µL) with 30 µL of LCMS grade methanol (ThermoFisher) in a 96-well microplate (Eppendorf). Plates were heat-sealed, vortexed for 5 min at 750 rpm, and centrifuged at 2000× *g* for 10 min at room temperature. The supernatant (10 µL) was carefully transferred to a 96-well plate, leaving behind the precipitated protein. The supernatant was further diluted with 10 µL of 100 mM ammonium formate, pH3. For hydrophilic interaction liquid chromatography (HILIC) analysis, the samples were diluted with 60 µL LCMS grade acetonitrile (ThermoFisher), whereas samples for C18 analysis were diluted with 60 µL water (GenPure ultrapure water system, Thermofisher). Each sample solution was transferred to 384-well microplate (Eppendorf) for LCMS analysis.

A total of 144 samples, plus quality control samples, were analyzed per batch per established operating procedures. Each batch was randomized, and the operator blinded; the ratio of cases to controls was equilibrated across each batch to mitigate bias. Matrix-matched reference-quality controls and batch-specific pooled quality controls were included in each batch.

### Appendix A.2. Analysis of Polyamines

Semi-quantitative measurement of plasma polyamines was conducted on Waters Acquity™ UPLC system with 2D column regeneration configuration (I-class and H-class) coupled to a Xevo G2-XS quadrupole time-of-flight (qTOF) mass spectrometer. Chromatographic separation was performed using HILIC (Acquity™ UPLC BEH amide, 100 Å, 1.7 µm 2.1 × 100 mm, Waters Corporation, Milford, CT, USA) and C18 (Acquity™ UPLC HSS T3, 100 Å, 1.8 µm, 2.1 × 100 mm, Water Corporation, Milford, U.S.A) columns at 45 °C.

Quaternary solvent system mobile phases were (A) 0.1% formic acid in water, (B) 0.1% formic acid in acetonitrile and (D) 100 mM ammonium formate, pH 3. Samples were separated using the following gradient profile: for the HILIC separation, a starting gradient of 95% B and 5% D was increased linearly to 70% A, 25% B, and 5% D over a 5 min period at 0.4 mL/min flow rate, followed by 1 min isocratic gradient at 100% A at 0.4 mL/min flow rate. For C18 separation, a chromatography gradient of was as follows: starting conditions, 100% A, with a linear increase to final conditions of 5% A, 95% B followed by isocratic gradient at 95% B, 5% D for 1 min.

A binary pump was used for column regeneration and equilibration. The solvent system mobile phases were (A1) 100 mM ammonium formate, pH 3, (A2) 0.1% formic in 2-propanol and (B1) 0.1% formic acid in acetonitrile. The HILIC column was stripped using 90% A2 for 5 min followed by 2 min equilibration using 100% B1 at 0.3 mL/min flowrate. Reverse phase C18 column regeneration was performed using 95% A1, 5% B1 for 2 min followed by column equilibration using 5% A1, 95% B1 for 5 min.

### Appendix A.3. Mass Spectrometry Data Acquisition

Mass spectrometry data were acquired in sensitivity and positive electrospray ionization mode within 50–1200 Da range. For the electrospray acquisition, the capillary voltage was set at 1.5 kV (positive), sample cone voltage 30 V, source temperature at 120 °C, cone gas flow 50 L/h and desolvation gas flow rate of 800 L/h with a scan time of 0.5 s in continuum mode. Leucine Enkephalin: 556.2771 Da (positive) was used for lockspray correction and scans were performed at 0.5 min. The injection volume for each sample was 6 µL, unless otherwise specified. The acquisition was carried out with instrument auto gain control to optimize instrument sensitivity over the samples acquisition time.

Pooled quality control samples were analyzed after a defined number of samples to assess replicate precision and allow LOESS correction by injection order. Additional data was captured using the MSe function for pooled quality control samples.

### Appendix A.4. Data Processing

Peak picking and retention time alignment of LC-MS and MSe data were performed using Progenesis QI software (Nonlinear, Waters). Data processing and peak annotations were performed using an in-house automated pipeline. Annotations for polyamines were determined by matching accurate mass and retention times using customized libraries created from authentic standards and by matching experimental tandem mass spectrometry data against the NIST MSMS, LipidBlast or HMDB v3 theoretical fragmentations. To correct for injection order drift, each feature was normalized using data from repeat injections of quality control samples collected every 10 injections throughout the run sequence. Measurement data were smoothed by locally weighted scatterplot smoothing (LOESS) signal correction (QC-RLSC) as previously described [10,11]. Values were reported as ratios relative to the median of historical quality control reference samples run with every analytical batch for the given analyte.

**Table A1:** Detailed information on cases and control subjects presenting with benign pelvic masses.

Condition	Description	Stage	GRADE	Set
Case	Mixed Broderline and Endometrioid Adenocarcinoma	2A	II	Test Set
Case	Serous carcinoma	2A	high	Test Set
Case	Clear Cell Carcinoma	2B	high	Test Set
Case	Serous Carcinoma	2B	high	Test Set
Case	Serous carcinoma	2B	low	Test Set
Case	Serous Carcinoma	2B	high	Test Set
Case	Clear Cell Carcinoma	2C2	high	Test Set
Case	Serous Carcinoma	4B	low	Test Set
Case	Clear cell carcinoma	I	high	Test Set
Case	clear cell carcinoma (CCC)	IA	high	Test Set
Case	Endometrioid adenocarcinoma	IA	II	Test Set
Case	Endometrioid adenocarcinoma	IA	I	Test Set
Case	Endometrioid Adenocarcinoma	IA	II	Test Set
Case	Endometrioid Adenocarcinoma	IA	II	Test Set
Case	Endometrioid adenocarcinoma	IA	II	Test Set
Case	Endometrioid adenocarcinoma	IA	I	Test Set
Case	Mixed: endometrioid carcinoma, serous carcinoma	IA	low	Test Set
Case	Mucinous Adenocarcinoma	IA	n/a	Test Set
Case	Mucinous carcinoma	IA	I	Test Set
Case	Mucinous Carcinoma	IA	high	Test Set
Case	Transitional cell carcinoma	IA	high	Test Set
Case	Serous carcinoma	IA	low	Test Set
Case	serous carcinoma	IA	high	Test Set
Case	endometrioid adenocarcinoma	IB	II	Test Set
Case	serous carcinoma	IB	high	Test Set
Case	Serous Carcinoma	IB	high	Test Set
Case	Serous Carcinoma in background of mixed epithelial borderline tumor	IB	low	Test Set
Case	Clear cell carcinoma	IC	high	Test Set
Case	Clear Cell Carcinoma	IC	high	Test Set
Case	clear cell carcinoma (CCC)	IC	n/a	Test Set
Case	Endometrioid Adenocarcinoma	IC	II	Test Set
Case	Endometrioid adenocarcinoma	IC	II	Test Set
Case	Serous carcinoma	IC	high	Test Set
Case	Serous carcinoma	IC	high	Test Set
Case	Serous Carcinoma	IC	high	Test Set
Case	Clear Cell Carcinoma	IC1	high	Test Set
Case	Carcinoma	IC1	n/a	Test Set
Case	Serous Carcinoma	IC2	high	Test Set
Case	Mixed: Serous Carcinoma & CCC	IC3	high	Test Set
Case	mucinous adenocarcinoma	II	n/a	Test Set
Case	mixed: undifferentiated carcinoma, serous carcinoma	IIA	high	Test Set
Case	mixed: endometrioid carcinoma, serous carcinoma	IIB	high	Test Set
Case	high serous carcinoma_III	III	high	Test Set
Case	high serous carcinoma_III	III	high	Test Set
Case	high serous carcinoma_III	III	high	Test Set
Case	high serous carcinoma_III	III	high	Test Set
Case	high serous carcinoma_III	III	high	Test Set
Case	high serous carcinoma_III	III	high	Test Set
Case	high serous carcinoma_III	III	high	Test Set
Case	high serous carcinoma_III	III	high	Test Set
Case	high serous carcinoma_III	III	high	Test Set
Case	Serous Carcinoma	IIIA	high	Test Set
Case	Serous carcinoma with minor endometrioid component	IIIA	high	Test Set
Case	high Serous Carcinoma_IIIB	IIIB	high	Test Set
Case	high Serous Carcinoma_”Advanced”	IIIC	high	Test Set
Case	high serous carcinoma_IIIC	IIIC	high	Test Set
Case	high serous carcinoma_IIIC	IIIC	high	Test Set
Case	high serous carcinoma_IIIC	IIIC	high	Test Set
Case	high serous carcinoma_IIIC	IIIC	high	Test Set
Case	high serous carcinoma_IIIC	IIIC	high	Test Set
Case	high Serous Carcinoma_IIIC	IIIC	high	Test Set
Case	high Serous Carcinoma_IIIC	IIIC	high	Test Set
Case	high Serous Carcinoma_IIIC	IIIC	high	Test Set
Case	high Serous Carcinoma_IIIC	IIIC	high	Test Set
Case	high Serous Carcinoma_IIIC	IIIC	high	Test Set
Case	high serous carcinoma_IIII	IIIC	high	Test Set
Case	high serous carcinoma_IIII	IIIC	high	Test Set
Case	high serous carcinoma_IIII	IIIC	high	Test Set
Case	high serous carcinoma_IIII	IIIC	high	Test Set
Case	high serous carcinoma_IIII	IIIC	high	Test Set
Case	high serous carcinoma_IIII	IIIC	high	Test Set
Case	high serous carcinoma_IIII	IIIC	high	Test Set
Case	high serous carcinoma_IIII	IIIC	high	Test Set
Case	high serous carcinoma_IIII	IIIC	high	Test Set
Case	high serous carcinoma_IIII	IIIC	high	Test Set
Case	high serous carcinoma_IIII	IIIC	high	Test Set
Case	high serous carcinoma_IIII	IIIC	high	Test Set
Case	high serous carcinoma_IIII	IIIC	high	Test Set
Case	high serous carcinoma_IIII	IIIC	high	Test Set
Case	high serous carcinoma_IIII	IIIC	high	Test Set
Case	high serous carcinoma_IIII	IIIC	high	Test Set
Case	high serous carcinoma_IIII	IIIC	high	Test Set
Case	high serous carcinoma_IIII	IIIC	high	Test Set
Case	high serous carcinoma_IIII	IIIC	high	Test Set
Case	high serous carcinoma_IIII	IIIC	high	Test Set
Case	high serous carcinoma_IIII	IIIC	high	Test Set
Case	high serous carcinoma_IIII	IIIC	high	Test Set
Case	high serous carcinoma_IIII	IIIC	high	Test Set
Case	high serous carcinoma_IIII	IIIC	high	Test Set
Case	high serous carcinoma_IIII	IIIC	high	Test Set
Case	high serous carcinoma_IIII	IIIC	high	Test Set
Case	high serous carcinoma_IIII	IIIC	high	Test Set
Case	high serous carcinoma_IIII	IIIC	high	Test Set
Case	high serous carcinoma_IIII	IIIC	high	Test Set
Case	high serous carcinoma_IIII	IIIC	high	Test Set
Case	high serous carcinoma_IIII	IIIC	high	Test Set
Case	high serous carcinoma_IIII	IIIC	high	Test Set
Case	high serous carcinoma_IIII	IIIC	high	Test Set
Case	high serous carcinoma_IIII	IIIC	high	Test Set
Case	high serous carcinoma_IIII	IIIC	high	Test Set
Case	high serous carcinoma_IIII	IIIC	high	Test Set
Case	high serous carcinoma_IIII	IIIC	high	Test Set
Case	high serous carcinoma_IIII	IIIC	high	Test Set
Case	high serous carcinoma_IIII	IIIC	high	Test Set
Case	high serous carcinoma_IIII	IIIC	high	Test Set
Case	high serous carcinoma_IIII	IIIC	high	Test Set
Case	high serous carcinoma_IV	IV	high	Test Set
Case	high serous carcinoma_IV	IV	high	Test Set
Case	high serous carcinoma_IV	IV	high	Test Set
Case	high serous carcinoma_IV	IV	high	Test Set
Case	high serous carcinoma_IV	IV	high	Test Set
Case	high serous carcinoma_IV	IV	high	Test Set
Case	high serous carcinoma_IV	IV	high	Test Set
Case	high serous carcinoma_IV	IV	high	Test Set
Case	high serous carcinoma_IV	IV	high	Test Set
Case	high Serous Carcinoma_IV	IV	high	Test Set
Case	serous carcinoma	I	high	Validation Set
Case	endometrioid adenocarcinoma	I	I	Validation Set
Case	mixed: endometrioid carcinoma, serous borderline tumor	I	II	Validation Set
Case	Serous Carcinoma	I	high	Validation Set
Case	serous carcinoma	I	high	Validation Set
Case	endometrioid adenocarcinoma	I	I	Validation Set
Case	Endometrioid Adenocarcinoma	IA	II	Validation Set
Case	serous carcinoma	IA	high	Validation Set
Case	mixed: endometrioid adenocarcinoma, clear cell carcinoma (CCC)	IA	high	Validation Set
Case	endometrioid adenocarcinoma	IA	I	Validation Set
Case	mucinous adenocarcinoma	IA	n/a	Validation Set
Case	Serous Mucinous Carcinoma	IA	n/a	Validation Set
Case	Serous Carcinoma	IA	high	Validation Set
Case	serous carcinoma	IA	high	Validation Set
Case	mucinous adenocarcinoma	IA	II	Validation Set
Case	Endometrioid Adenocarcinoma, Mucinous Carcinoma, Clear Cell Carcinoma	IA	Mixed high and low	Validation Set
Case	mixed: papillary serous carcinoma, endometrioid carcinoma	IA	high	Validation Set
Case	clear cell carcinoma (CCC)	IA	high	Validation Set
Case	endometrioid adenocarcinoma	IA	high	Validation Set
Case	serous adenocarcinoma	IA	high	Validation Set
Case	serous carcinoma	IA	high	Validation Set
Case	clear cell carcinoma (CCC)	IA	high	Validation Set
Case	endometrioid adenocarcinoma	IB	II	Validation Set
Case	carcinoma	IB	high	Validation Set
Case	Mixed Serous and Endometrioid Carcinoma	IC	high	Validation Set
Case	endometrioid adenocarcinoma	IC	II	Validation Set
Case	endometrioid adenocarcinoma	IC	II	Validation Set
Case	endometrioid adenocarcinoma	IC	III	Validation Set
Case	Clear Cell Carcinoma	IC	high	Validation Set
Case	endometrioid adenocarcinoma	IC	I	Validation Set
Case	mixed: endometrioid carcinoma, ovarian carcinoma, peritoneal carcinoma	IC	II	Validation Set
Case	mucinous carcinoma	IC	low	Validation Set
Case	serous carcinoma	IC	low	Validation Set
Case	serous carcinoma	IC	high	Validation Set
Case	mixed: endometrioid carcinoma, clear cell carcinoma (CCC)	IC	high	Validation Set
Case	mucinous adenocarcinoma	IC	I	Validation Set
Case	mucinous carcinoma	IC	n/a	Validation Set
Case	Mucinous Carcinoma	IC	II	Validation Set
Case	endometrioid adenocarcinoma	II	II	Validation Set
Case	Endometroid Adenocarcinoma	II	n/a	Validation Set
Case	mixed: transitional carcinoma, squamous cell carcinoma	II	high	Validation Set
Case	Serous Carcinoma	II	high	Validation Set
Case	serous carcinoma	IIA	high	Validation Set
Case	endometrioid adenocarcinoma	IIA	III	Validation Set
Case	Serous Carcinoma	IIA	High	Validation Set
Case	mixed: undifferentiated carcinoma, serous carcinoma	IIA	high	Validation Set
Case	mixed: endometrioid carcinoma, serous carcinoma	IIB	high	Validation Set
Case	Serous Carcinoma, Adenocarcinoma	IIB	High	Validation Set
Case	endometrioid adenocarcinoma	IIB	II	Validation Set
Case	serous carcinoma	IIB	low	Validation Set
Case	mixed: serous carcinoma, endometrioid carcinoma	IIB	high	Validation Set
Case	serous carcinoma	IIB	high	Validation Set
Case	mixed: mucinous endometrioid carcinoma, clear cell carcinoma (CCC)	IIB	high	Validation Set
Case	serous carcinoma	IIB	high	Validation Set
Case	Serous Carcinoma	IIB	high	Validation Set
Case	Serous Carcinoma	IIB	high	Validation Set
Case	serous carcinoma	IIB	high	Validation Set
Case	serous carcinoma	IIC	high	Validation Set
Case	Serous Carcinoma	IIC	high	Validation Set
Case	Clear Cell Carcinoma	IIC	n/a	Validation Set
Case	Serous Carcinoma	IIC	Low	Validation Set
Benign Pelvic Mass	Serous adenofibroma	-	-	Test Set
Benign Pelvic Mass	Serous Cystadoma	-	-	Test Set
Benign Pelvic Mass	Mucinous Cystadenoma	-	-	Test Set
Benign Pelvic Mass	Mucinous Cystadenofribroma	-	-	Test Set
Benign Pelvic Mass	Endometriotic Cyst	-	-	Test Set
Benign Pelvic Mass	mucinous neoplasm	-	-	Test Set
Benign Pelvic Mass	serous cystadenomas	-	-	Test Set
Benign Pelvic Mass	Fibroma	-	-	Test Set
Benign Pelvic Mass	serous cystadenofibroma	-	-	Test Set
Benign Pelvic Mass	serous cystadenoma	-	-	Test Set
Benign Pelvic Mass	Mucinous Cystadenoma	-	-	Test Set
Benign Pelvic Mass	Fibroma	-	-	Test Set
Benign Pelvic Mass	Serous cystadenoma	-	-	Test Set
Benign Pelvic Mass	Cyst & Rete Cystadonoma	-	-	Test Set
Benign Pelvic Mass	Peritoneal Inclusion Cysts, Follicular Cysts, Corpus Luteal Cysts	-	-	Test Set
Benign Pelvic Mass	serous cystadenofibroma	-	-	Test Set
Benign Pelvic Mass	endometrioma, endometriosis	-	-	Test Set
Benign Pelvic Mass	cystadenoma and severe pelvic adhesive disease	-	-	Test Set
Benign Pelvic Mass	cystadenofibroma	-	-	Test Set
Benign Pelvic Mass	Endometriotic cyst	-	-	Test Set
Benign Pelvic Mass	cyst	-	-	Test Set
Benign Pelvic Mass	ovarian mass	-	-	Test Set
Benign Pelvic Mass	Mucinous Cystadenoma	-	-	Test Set
Benign Pelvic Mass	mucinous cystadenoma	-	-	Test Set
Benign Pelvic Mass	fibroma, cysts	-	-	Test Set
Benign Pelvic Mass	mucinous cystadenoma	-	-	Test Set
Benign Pelvic Mass	mucinous cystadenoma, intestinal	-	-	Test Set
Benign Pelvic Mass	Mucinous cystadenoma	-	-	Test Set
Benign Pelvic Mass	bilateral endometriomas	-	-	Test Set
Benign Pelvic Mass	Mucinous Cystadenoma	-	-	Test Set
Benign Pelvic Mass	Endometriotic Cyst	-	-	Test Set
Benign Pelvic Mass	cyst	-	-	Test Set
Benign Pelvic Mass	cystadenoma	-	-	Test Set
Benign Pelvic Mass	Mucinous cystadenoma	-	-	Test Set
Benign Pelvic Mass	cyst	-	-	Test Set
Benign Pelvic Mass	Endometriotic cyst	-	-	Test Set
Benign Pelvic Mass	Cysts	-	-	Test Set
Benign Pelvic Mass	serous ovarian cystadenofibroma of LOV	-	-	Test Set
Benign Pelvic Mass	benign mass	-	-	Test Set
Benign Pelvic Mass	Luteal Cyst	-	-	Test Set
Benign Pelvic Mass	mucinous cystadenoma	-	-	Test Set
Benign Pelvic Mass	serous cystadenoma	-	-	Test Set
Benign Pelvic Mass	Serous Cystadenofibroma	-	-	Test Set
Benign Pelvic Mass	Serous Cystadenofibroma	-	-	Test Set
Benign Pelvic Mass	serous cyst	-	-	Test Set
Benign Pelvic Mass	cystadenoma	-	-	Test Set
Benign Pelvic Mass	Mucinous Cystadenofribroma	-	-	Test Set
Benign Pelvic Mass	mucinous cystadenoma	-	-	Test Set
Benign Pelvic Mass	serous cystadenoma	-	-	Test Set
Benign Pelvic Mass	Cysts	-	-	Test Set
Benign Pelvic Mass	mucinous cystadenoma	-	-	Test Set
Benign Pelvic Mass	endometrioma	-	-	Test Set
Benign Pelvic Mass	Fibroma	-	-	Test Set
Benign Pelvic Mass	Sertoli-Leydig cell	-	-	Test Set
Benign Pelvic Mass	serous cystadenofibroma	-	-	Test Set
Benign Pelvic Mass	endometrioisis, atypia	-	-	Test Set
Benign Pelvic Mass	mucinous cystadenoma	-	-	Test Set
Benign Pelvic Mass	Benign Luteal Cyst	-	-	Test Set
Benign Pelvic Mass	Endometriosis	-	-	Test Set
Benign Pelvic Mass	Cystadenofibroma	-	-	Test Set
Benign Pelvic Mass	Cyst	-	-	Test Set
Benign Pelvic Mass	cysts	-	-	Test Set
Benign Pelvic Mass	(LOV) fibroma	-	-	Test Set
Benign Pelvic Mass	endometrioic cysts	-	-	Test Set
Benign Pelvic Mass	serous cystadenofibroma	-	-	Test Set
Benign Pelvic Mass	Serous Cystadenofibroma	-	-	Test Set
Benign Pelvic Mass	Mucinous Cystadenona	-	-	Test Set
Benign Pelvic Mass	Mucinous Cystadenoma	-	-	Test Set
Benign Pelvic Mass	corpus albicanocyst, fibrosis	-	-	Test Set
Benign Pelvic Mass	fibroma	-	-	Test Set
Benign Pelvic Mass	brenner tumor	-	-	Test Set
Benign Pelvic Mass	fibrothecoma of ROV	-	-	Test Set

**Table A2:** Polyamines detected and measured by liquid chromatography mass spectrometry.

Metabolite	Adduct	m/z	Retention Time (min)	Assay †
Acetylspermidine	[M + H] +	188.1751	3.85	P_HA
Diacetylspermidine	[M + H] +	230.1858	3.11	P_HA
Diacetylspermine	[M + H] +	287.2418	3.72	P_HA
N-(3-acetamidopropyl)pyrrolidin-2-one	[M + H] +	185.1277	2.12	P_CA

† P: electrospray ionization positive mode acquisition; HA- HILIC acidic; CA- C18 acidic.

**Table A3:** Coefficient of variation (CV) values for measured polyamines in pooled quality control samples.

Coefficient of Variation Values for Measured Polyamines	Coefficient of Variation (CV) Values					
	Test Set				Validation Set	
	Batch 1		Batch 2		Batch 3	
	HQC	PQC	HQC	PQC	HQC	PQC
DAS	21.26	4.52	5.57	3.25	11.68	5.29
AcSpmd	4.61	3.25	5.19	2.02	21.29	2.58
DiAcspmd	6.56	6.66	5.61	4.08	15.21	4.86
N3AP	5.61	2.96	3.64	4.74	3.5	1.85

Abbreviation: DAS- diacetylspermine; AcSpmd- Acetylspermidine; DiAcspmd- Diacetylspermidine; N3AP- N-(3-acetamidopropyl)pyrrolidin-2-one.

**Table A4:** Classification performances of plasma polyamines in distinguishing ovarian cancer cases from controls in the Test Set.

	**All OvCa Cases (*n* = 116) vs.** **Healthy Controls (*n* = 71)**					**Early Stage OvCa Cases (*n* = 41) vs.** **Healthy Controls (*n* = 71)**					**Late Stage OvCa Cases (*n* = 75) vs.** **Healthy Controls (*n* = 71)**				
Metabolite	AUC	95% CI	*p* †	Sens @ 95% Spec	Spec @ 95% Sens	AUC	95% CI	*p* †	Sens @ 95% Spec	Spec @ 95% Sens	AUC	95% CI	*p* †	Sens @ 95% Spec	Spec @ 95% Sens
Diacetylspermine	0.88	0.84–0.93	<0.001	47.4	59.2	0.82	0.74–0.90	<0.001	43.9	43.7	0.92	0.88–0.96	<0.001	49.3	73.2
Diacetylspermidine	0.74	0.66–0.82	<0.001	8.6	35.2	0.76	0.67–0.85	<0.001	22.0	31.0	0.73	0.64–0.82	<0.001	1.3	50.7
Acetylspermidine	0.76	0.69–0.83	<0.001	29.3	19.7	0.70	0.59–0.81	<0.001	29.3	5.6	0.79	0.72–0.87	<0.001	29.3	38.0
N3AP	0.75	0.67–0.82	<0.001	18.1	21.1	0.72	0.62–0.82	<0.001	19.5	21.1	0.76	0.69–0.84	<0.001	17.3	32.4
	**All OvCa Cases (*n* = 116) vs.** **Subjects with Benign Cysts (*n* = 72)**					**Early Stage OvCa Cases (*n* = 41) vs.** **Subjects with Benign Cysts (*n* = 72)**					**Late Stage OvCa Cases (*n* = 75) vs.** **Subjects with Benign Cysts (*n* = 72)**				
Metabolite	AUC	95% CI	*p* †	Sens @ 95% Spec	Spec @ 95% Sens	AUC	95% CI	*p* †	Sens @ 95% Spec	Spec @ 95% Sens	AUC	95% CI	*p* †	Sens @ 95% Spec	Spec @ 95% Sens
Diacetylspermine	0.78	0.72–0.85	<0.001	28.4	34.7	0.70	0.60–0.81	< 0.001	24.4	22.2	0.83	0.76–0.89	<0.001	30.7	56.9
Diacetylspermidine	0.52	0.43–0.60	0.73	0	2.8	0.58	0.46–0.70	0.17	17.1	5.6	0.57	0.47–0.66	0.17	0	18.1
Acetylspermidine	0.53	0.45–0.61	0.50	17.2	0	0.50	0.38–0.63	0.98	22.0	0	0.55	0.45–0.64	0.33	16.0	2.8
N3AP	0.58	0.50–0.66	0.06	19.0	1.4	0.55	0.43–0.67	0.38	22.0	1.4	0.60	0.51–0.69	0.04	17.3	5.6

† Significance was determined by 2-sided Wilcoxon rank sum test.

**Table A5:** Classification performance of plasma polyamines in distinguishing serous and non-serous cases from controls in the Test Set.

	**All Serous Cases (*n* = 91) vs. Healthy Controls (*n* = 71)**					**All Serous Cases (*n* = 91) vs. Subjects with Benign Cysts (*n* = 72)**				
Metabolite	AUC	95% CI	*p* †	Sens @ 95% Spec	Spec @ 95% Sens	AUC	95% CI	*p* †	Sens @ 95% Spec	Spec @ 95% Sens
Diacetylspermine	0.9	0.85–0.95	<0.001	47.3	69	0.8	0.74–0.87	<0.001	28.6	47.2
Diacetylspermidine	0.73	0.64–0.81	<0.001	3.3	39.4	0.55	0.46–0.64	0.28	0	9.7
Acetylspermidine	0.77	0.69–0.84	<0.001	29.7	23.9	0.53	0.44–0.62	0.5	16.5	0
N3AP	0.75	0.68–0.83	<0.001	17.6	32.4	0.58	0.50–0.67	0.07	17.6	5.6
	Early Stage Serous Cases (*n* = 16) vs. Healthy Controls (*n* = 71)					Early Stage Serous Cases (*n* = 16) vs. Subjects with Benign Disease (*n* = 72)				
Metabolite	AUC	95% CI	*p* †	Sens @ 95% Spec	Spec @ 95% Sens	AUC	95% CI	*p* †	Sens @ 95% Spec	Spec @ 95% Sens
Diacetylspermine	0.81	0.69–0.94	<0.001	37.5	4.2	0.7	0.56–0.84	0.01	18.8	5.6
Diacetylspermidine	0.74	0.62–0.87	0.003	12.5	25.4	0.47	0.30–0.64	0.74	0	2.8
Acetylspermidine	0.66	0.49–0.83	0.049	31.3	4.2	0.54	0.34–0.75	0.58	31.3	0
N3AP	0.7	0.56–0.84	0.01	18.8	18.3	0.51	0.34–0.68	0.94	18.8	0
	Late Stage Serous Cases (*n* = 75) vs. Healthy Controls (*n* = 71)					Late Stage Serous Cases (*n* = 75) vs. Subjects with Benign Disease (*n* = 72)				
Metabolite	AUC	95% CI	*p* †	Sens @ 95% Spec	Spec @ 95% Sens	AUC	95% CI	*p* †	Sens @ 95% Spec	Spec @ 95% Sens
Diacetylspermine	0.92	0.88–0.96	<0.001	49.3	73.2	0.83	0.76–0.89	<0.001	30.7	56.9
Diacetylspermidine	0.73	0.64–0.82	<0.001	1.3	50.7	0.57	0.47–0.66	0.17	0	18.1
Acetylspermidine	0.79	0.72–0.87	<0.001	29.3	38	0.55	0.45–0.64	0.33	16	2.8
N3AP	0.76	0.69–0.84	<0.001	17.3	32.4	0.6	0.51–0.69	0.04	17.3	5.6
	Non-Serous Cases (*n* = 25) vs. Healthy Controls (*n* = 71)					Non-Serous Cases (*n* = 25) vs. Subjects with Benign Disease (*n* = 72)				
Metabolite	AUC	95% CI	*p* †	Sens @ 95% Spec	Spec @ 95% Sens	AUC	95% CI	*p* †	Sens @ 95% Spec	Spec @ 95% Sens
Diacetylspermine	0.83	0.73–0.92	<0.001	48	43.7	0.71	0.58–0.83	0.002	28	22.2
Diacetylspermidine	0.77	0.67–0.88	<0.001	28,0	31	0.61	0.46–0.76	0.1	24	5.6
Acetylspermidine	0.72	0.59–0.85	0.001	28	5.6	0.53	0.37–0.68	0.71	20	0
N3AP	0.73	0.61–0.85	<0.001	20	21.1	0.58	0.43–0.73	0.25	24	1.4

† Significance was determined by 2-sided Wilcoxon rank sum test.

**Table A6:** Sensitivity of the 3-marker panel and CA125 alone at specificity cutoff values of 99%, 98.5% and 97% in the Test Set and Validation Set.

	**Test Set**		
**Cutoffs**	**DAS + N3AP + CA125**	**CA125**	**McNemar Exact Test 1-sided *p*-value**
Sensivitiy @ 99% Specificity	46.3	24.3	0.006
Sensivitiy @ 98.5% Specificity	48.8	34.1	0.04
Sensivitiy @ 97% Specificity	48.8	39.2	0.17
	Validation Set		
Cutoffs	DAS + N3AP + CA125	CA125	McNemar Exact Test 1-sided *p*-value
Sensivitiy @ 99% Specificity	73.7	62.2	0.02
Sensivitiy @ 98.5% Specificity	78.6	81.9	0.37
Sensivitiy @ 97% Specificity	83.6	86.8	0.38

**Table A7:** Classification performance of plasma polyamines in distinguishing serous or non-serous cases from controls in the Validation Set.

	All Cases (*n* = 61) vs. Controls (*n* = 71)					Serous Cases (*n* = 28) vs. Controls (*n* = 71)					Non-Serous Cases (*n* = 33) vs. Controls (*n* = 71)				
Metabolite	AUC	95% CI	*p* †	Sens @ 95% Spec	Spec @ 95% Sens	AUC	95% CI	*p* †	Sens @ 95% Spec	Spec @ 95% Sens	AUC	95% CI	*p* †	Sens @ 95% Spec	Spec @ 95% Sens
Diacetylspermine	0.84	0.77–0.91	<0.001	52.5	33.8	0.84	0.75–0.93	<0.001	46.4	33.8	0.84	0.75–0.92	<0.001	57.6	23.9
Diacetylspermidine	0.57	0.47–0.67	0.18	13.1	1.4	0.56	0.42–0.70	0.33	10.7	0	0.57	0.44–0.70	0.25	15.2	1.4
Acetylspermidine	0.67	0.57–0.77	<0.001	27.9	2.8	0.64	0.50–0.78	0.03	25.0	2.8	0.69	0.57–0.81	0.002	30.3	2.8
N3AP	0.59	0.49–0.69	0.08	18.0	4.2	0.58	0.44–0.72	0.21	17.9	4.2	0.59	0.46–0.72	0.12	18.2	2.8

† Significance was determined by 2-sided Wilcoxon rank sum test.
